# Wound healing of human embryonic stem cell-derived retinal pigment epithelial cells is affected by maturation stage

**DOI:** 10.1186/s12938-018-0535-z

**Published:** 2018-07-31

**Authors:** Amna E. Abu Khamidakh, Alejandra Rodriguez-Martinez, Kai Kaarniranta, Anne Kallioniemi, Heli Skottman, Jari Hyttinen, Kati Juuti-Uusitalo

**Affiliations:** 1 Faculty of Biomedical Sciences and Engineering, BioMediTech, Tampere University of Technology, Arvo Ylpön Katu 34, Tampere, Finland; 2 Faculty of Medical and Life Sciences, BioMediTech, University of Tampere, Arvo Ylpön Katu 34, Tampere, Finland; 3 Department of Ophthalmology, Institute of Clinical Medicine, University of Eastern Finland, Kuopio, Finland; 4 Department of Ophthalmology, Kuopio University Hospital, Kuopio, Finland

**Keywords:** Image analysis, hESC-RPE, RPE, Cell maturation, Wound healing, Spontaneous [Ca^2+^]_i_ increases, Mechanical stimulation, Ca^2+^ waves, Mechanically induced intercellular Ca^2+^ waves

## Abstract

**Background:**

Wound healing of retinal pigment epithelium (RPE) is a complex process that may take place in common age-related macular degeneration eye disease. The purpose of this study was to evaluate whether wounding and wound healing has an effect on Ca^2+^ dynamics in human embryonic stem cell (hESC)-RPEs cultured different periods of time.

**Methods:**

The 9-day-cultured or 28-day-cultured hESC-RPEs from two different cell lines were wounded and the dynamics of spontaneous and mechanically induced intracellular Ca^2+^ activity was measured with live-cell Ca^2+^ imaging either immediately or 7 days after wounding. The healing time and speed were analyzed with time-lapse bright field microscopy. The Ca^2+^ activity and healing speed were analysed with image analysis. In addition the extracellular matrix deposition was assessed with confocal microscopy.

**Results:**

The Ca^2+^ dynamics in hESC-RPE monolayers differed depending on the culture time: 9-day-cultured cells had higher number of cells with spontaneous Ca^2+^ activity close to freshly wounded edge compared to control areas, whereas in 28-day-cultured cells there was no difference in wounded and control areas. The 28-day-cultured, wounded and 7-day-healed hESC-RPEs produced wide-spreading intercellular Ca^2+^ waves upon mechanical stimulation, while in controls propagation was restricted. Most importantly, both wave spreading and spontaneous Ca^2+^ activity of cells within the healed area, as well as the cell morphology of 28-day-cultured, wounded and thereafter 7-day-healed areas resembled the 9-day-cultured hESC-RPEs.

**Conclusions:**

This acquired knowledge about Ca^2+^ dynamics of wounded hESC-RPE monolayers is important for understanding the dynamics of RPE wound healing, and could offer a reliable functionality test for RPE cells. The data presented in here suggests that assessment of Ca^2+^ dynamics analysed with image analysis could be used as a reliable non-invasive functionality test for RPE cells.

**Electronic supplementary material:**

The online version of this article (10.1186/s12938-018-0535-z) contains supplementary material, which is available to authorized users.

## Background

The retinal pigment epithelial (RPE) cells form a monolayer of tightly packed cells which lay between neurosensory retina and the choroid [1]. The RPEs absorb stray light, transport the nutrients from the choroidal side to neural retina, regulate visual cycle and secrete various trophic factors maintaining retinal homeostasis [2].

In pathological conditions RPE dysfunction may lead to the cell layer disruption and choroidal neovascularization as observed in wet age-related macular degeneration (AMD) [3]. AMD is the leading cause of irreversible blindness among the elderly in Western countries. AMD can be divided into dry and wet forms with ~ 80 and ~ 20% prevalence, respectively. No efficient treatment exists today for dry AMD. Wet AMD development is strongly associated with the vascular endothelial growth factor (VEGF)-derived aberrant blood vessels sprout from the choroidal capillaries that penetrate through the Bruch’s membrane and RPE into subretinal space. If left untreated, choroidal neovascularization evokes hemorrhage, retinal edema, and increased damage to retinal cells, fibrosis and permanent visual loss due to the undergoing wound healing process. VEGF is the principal therapy target for inhibiting the detrimental neovascularization process, i.e. by intravitreal administration of ranibizumab or bevacizumab or VEGF Trap [4]. Also the intravitreal injection itself may be traumatic and lead to RPE tear and wound healing.

The functionality of RPE after wounding has been assessed in various animal models, for example, rabbit [5, 6], chick [7], porcine [8], bovine RPE [9] and in donor human RPE cells, [10, 11] and human ESC-RPE cells [12]. These studies demonstrated that RPE cells on the wound edge undergo dedifferentiation, changing their phenotype towards the phenotype of migrating cells: the cells evolve cytoskeletal reorganization and partially or completely lose their pigmentation [5, 6, 8, 13].

Ca^2+^ is an important second messenger which mediates vital cellular functions from cell proliferation to cell death. The calcium transients triggered after ATP stimulation are used as a functionality assessment for stem cell derived RPE cells [14, 15]. In addition, Ca^2+^ is known to play an important role in the wound healing process [16].

Immediately following tissue wounding, intracellular Ca^2+^ concentration ([Ca^2+^]_i_) increases in the cells located at the edge of the wound. This [Ca^2+^]_i_ increase then spreads to neighboring cells, forming so-called intercellular Ca^2+^ wave, which activates undamaged cells, triggers reorganization of cell junctions and coordinates cell motility [17, 18].

In RPE cells, Ca^2+^ controls important physiological processes, such as dark/light adaptation of photoreceptors, phagocytosis, trans-epithelial transport of fluid and ions, secretion of growth factors, as well as and differentiation [19].

RPE cells exhibit spontaneous [Ca^2+^]_i_ increases that are important for communication with retina [20, 21]. We have recently shown that human RPE cells have distinct spontaneous Ca^2+^ activity at different maturation stages. The immature RPEs, where the majority of the cells have fusiform morphology and low level of pigmentation, have lower amount of cells with spontaneous [Ca^2+^]_i_ increases compared with more mature, cobblestone-shaped, highly-pigmented cells [22].

It has been demonstrated that upon mechanical stimulation of a single cell within a monolayer, RPE cells trigger a Ca^2+^ wave that originates from the stimulated cell and propagates to neighboring cells [23–25]. Previously, we found that, similarly to spontaneous [Ca^2+^]_i_ increases, the properties of these Ca^2+^ waves strongly depend on RPE maturation stage. In immature cells, the mechanically induced Ca^2+^ waves spread far away from the site of stimulation, while in more mature RPE these waves propagate to minute number of cells surrounding the site of stimulation [22].

In wounded monolayers, the cells located in the vicinity of the denuded area are important for triggering a wound healing process [17, 18]. Furthermore, in the healed wounds, the RPE cells transiently change their morphology towards a less mature phenotype [5, 12, 13]. Thus, we hypothesize that these changes are reflected in Ca^2+^ dynamics of cells located around the wounded area and inside of healed wounds.

In this paper, we utilized image analysis tool to assess whether wounding and wound healing has effects on Ca^2+^ dynamics in hESC-RPEs cultured different periods of time. In addition, the effect of culture time on wound speed and time of hESC-RPE cells was evaluated. The knowledge of Ca^2+^ dynamics in freshly wounded and healed RPE monolayers can shed light on the mechanism of wound healing, and this possibly can be applied to understand the healing process of RPEsin the pathology of wet AMD or its treatment modalities.

## Methods

### Differentiation of hESC-RPE cells

Two hESC lines, Regea08/023 and Regea08/017, that were derived and characterized in our laboratory as described previously [26], were used in this study. The undifferentiated hESC lines were cultured on top of mitotically inactivated, mitomycin (10 μg/ml, Sigma-Aldrich, St. Louis, USA) treated, human foreskin fibroblasts feeder cells (CRL-2429TM, ATCC, Manassas, VA, USA) [27].

The hESCs were differentiated towards RPE in a RPEbasic medium as cell aggregates for 56–93 days. The selection of pigmented areas from floating aggregates was done manually, isolated areas were dissociated with 1× Trypsin–EDTA (Lonza, Walkersville, USA), plated with methods described previously, [28] and cultured in average for 85 days (ranging from 28 to 130) to expand amount of cells.

### Cell culturing for experiments

When the amount of cells was sufficient, the hESC-RPE cells were dissociated with 1× Trypsin–EDTA again and plated on top of Ormocomp-treated (Micro Resist Technology GmbH, Germany) [29], ColIV (Sigma-Aldrich)-coated coverslips (7 mm in diameter; Thermo Fisher Scientific, Inc., Leicestershire, UK) at a density of 10^5^ cells/cm^2^. Cells were cultured for a period of 9 ± 1, 16 ± 1, 28 ± 1, or 35 ± 1 days in RPEbasic medium, during which the medium was replenished thrice a week.

### RT-PCR analysis

The expression of RPE specific genes, the microphthalmia-associated transcription factor (*MITF*), bestrophin (*BEST*) and tyrosinase were assessed with reverse transcription–polymerase chain reaction (RT-PCR). A housekeeping gene, glyceraldehyde 3-phosphate dehydrogenase (GAPDH) was used as an internal control. The total RNA was extracted from hESC-RPE cells after 9, 16, 28, and 35 days of culture with a NucleoSpin XS-kit (Macherey–Nagel, GmbH & Co, Duren, Germany) according to the manufacturer’s instructions. The RT-PCR protocol was carried out as previously reported [28]. The primer sequences, annealing temperatures and product sizes are presented in Table 1.

**Table 1:** Reverse-transcriptase-PCR primer sequences, product lengths (bp) and used annealing temperatures

Gene	Forward	Reverse	bp	Tm
GAPDH	GTTCGACAGTCAGCCGCATC	GGAATTTGCCATGGGTGGA	229	55
MITF	AAGTCCTGAGCTTGCCATGT	GGCAGACCTTGGTTTCCATA	352	52
BEST	GAATTTGCAGGTGTCCCTGT	ATCAGGAGGACGAGGAGGAT	214	60
Tyrosinase	TGC CAA CGA TCC TAT CTT CC	GAC ACA GCA AGC TCA CAA GC	52	316

Primer sequences (5′ > 3′)

### Quantitative RT-PCR analysis

The expression of RPE specific genes was further analysed with quantitative RT-PCR by utilising TaqMan^®^ Gene Expression Assays (Applied Biosystems Inc.) with FAM-labeled primers for retinal pigment epithelium specific protein 65 kDa (RPE65, hs01071462_m1) and receptor tyrosine kinase MerTK (MERTK, hs00179024_m1). The glyceraldehyde 3-phosphate dehydrogenase (GAPDH) (Hs99999905_m1) was used as an endogenous control. Samples and negative controls (no template) were run as triplicate reactions using the 7300 Real-time PCR system (Applied Biosystems Inc.) similarly as in Sorkio and others [30]. Thereafter the relative quantification of each gene was calculated with the 2^−ΔΔCt^ method [31] using GAPDH expression level of 9-day cultured Regea08/017 as the calibrator for both the 9- and 28-day-cultured Regea08/017, and the GAPDH expression level of 9-day Regea08/023 as the calibrator for both the 9- and 28-day-cultured Regea08/023. For both calibrators the fold change equals 1.

### Immunofluorescence staining and quantification of cell area

Localization of tight junction-forming Zona Occludens Protein 1 (ZO-1), marker of proliferative cells (Ki67), extracellular matrix forming collagen I (ColI) and laminin (Lam) were assessed with indirect immunofluorescence labeling as described previously [25]. Briefly, the hESC-RPE cells on Ormocomp^®^ coated coverslips were rinsed in PBS thrice and fixed in 4% paraformaldehyde (Sigma-Aldrich) in 1× PBS (Lonza Group Ltd) for 10 min. Then, the cells were again rinsed with PBS 3 times and permeabilized for 10 min with 0.01% Triton X (Sigma-Aldrich) in PBS at RT. Cells were washed thrice in PBS, and unspecific antibody binding sites were blocked with 3% bovine serum albumin (BSA; Sigma-Aldrich) in PBS at RT for 1 h. Next, the cells were incubated with primary antibodies (see Table 2) dissolved in 0.5% BSA-PBS for 1 h at RT, and thereafter were washed thrice with 1× PBS. Cells were then incubated with secondary antibodies (see Table 2) diluted in 0.5% BSA-PBS for 1.5 h at RT. Finally, the cells were washed thrice with PBS and mounted with Vectashield mount with 4′,6′diamidino-2-phenylidole (DAPI [Vector Laboratories Inc., Burlingame, CA, USA]). Samples were visualized under Zeiss LSM 700 confocal microscope (Zeiss, Jena, Germany) with a 63× oil immersion objective or under Olympus IX51 fluorescence microscope with a 20× air objective. Confocal images were edited with ZEN 2012 black (Zeiss).

**Table 2:** Primary and secondary antibodies for immunofluorescence staining

Antibodies	Dilution	Manufacturer
I Ab: Mouse-anti-human ZO-1	1:250	Life Technologies, Carlsbad, USA
I Ab: Ki67	1:500	Millipore, Darmstadt, Germany
I Ab: Laminin	1:100	Abcam, Cambridge, UK
I Ab: ColI	1:250	Abcam, Cambridge, UK
II Ab: Alexa Fluor 488 donkey-anti-rabbit	1:800	Molecular Probes, Leicestershire, UK
II Ab: Alexa Fluor 568 donkey-anti-mouse	1:800	Molecular Probes, Leicestershire, UK

*I Ab* primary antibody, *II Ab* secondary antibody

The differences in cell shapes were estimated from cell areas from immunofluorescence images with ZO-1 labeling. In Fiji, the cell borders of 100 randomly selected cells were defined manually for 9-, 16-, 28-, and 35-day-cultured non-wounded cells and inside 7-day-healed wounds of the cells wounded on day 28 of culture. Individual cell areas were calculated with a standard Fiji measurement option. The presented data are combined from Regea08/017 and Regea08/023 hESC-RPEs.

### Wounding of hESC-RPEs

The wounding of day 9- or 28-day cultured hESC-RPE monolayers were done mechanically by performing a linear scratch with a plastic 10 μl pipette tip. Although the person who did the injury was always the same and tried to perform it similarly, with same speed and pressure, there might be variation due to the manual handling. When Ca^2+^ dynamics immediately after wounding was about to be assessed, the cells were allowed to rest for 15 min after wounding, before the actual experiments were started, thus the samples are abbreviated as 9 days + 15 min or 28 days + 15 min samples. When wound healing was evaluated, the cellular monolayers were allowed to grow for 7–8 days prior to the experiments (abbreviated as 9 days + 7 days or 28 days + 7 days samples). Wound healing process was tracked with time-lapse microscopy in Nikon BioStation CT (Nikon, Nikon Instruments Europe BV, Netherlands). There, the cells were cultured at 37 °C and 5% CO_2_, and phase contrast images of wounded areas were automatically recorded every 1–3 h during the healing period with a 10× objective. The medium was replenished thrice a week.

Wound healing speed and time were assessed only from the samples without visible ColIV coating damage. The speed of healing was calculated by analyzing the wound size at the beginning of the assay (in pixels) divided by full healing time in hours. Wound healing time was estimated as time in hours needed for the hESC-RPEs to completely cover the cell-free lane with the cells. The percentage of healed samples was assessed from samples with both damaged and intact ColIV coating. The presented data are combined from Regea08/017 and Regea08/023 hESC-RPEs.

### Ca^2+^ imaging

The Ca^2+^ dynamics in cells around fresh wounds (9 days + 15 min and 28 days + 15 min samples) and in healed cultures (9 days + 7 days and 28 days + 7 days samples) was evaluated with live-cell Ca^2+^ imaging. Two types of measurements were performed on control and wounded hESC-RPEs: first the spontaneous Ca^2+^ activity was recorded from unstimulated cells at least for 5 min time; second, intercellular Ca^2+^ waves spreading was recorded after mechanical stimulation again at least for 5 min time.

All Ca^2+^ imaging recordings were performed in HBSS medium, containing 5 mM glucose, 20 mM HEPES, 0.44 mM KH_2_PO_4_, 137 mM NaCl, 5 mM KCl, 4.2 mM NaHCO_3_, 1.2 mM MgCl_2_, and 2 mM CaCl_2_. pH was adjusted to 7.37 with 3 M NaOH; osmolarity was adjusted to 335 ± 5 mOsm/kg with sucrose. All components were from Sigma-Aldrich.

For the live-cell Ca^2+^ imaging recordings hESC-RPEs were loaded with Fluo-4-acetoxymethyl ester (Fluo-4 AM; 4 μM, in 1 h incubation at room temperature (RT), Life Technologies, Carlsbad, USA). Next, the cells were washed with HBSS thrice. The coverslips with Fluo-4 AM loaded cells were then mounted on a perfusion chamber (Warner Instruments, Hamden, USA) and filled with HBSS. The recording of the fluorescence signal was done under an Olympus IX51 fluorescence microscope (Olympus, Tokyo, Japan) with ANDOR iXion 885 camera (Oxford Instruments, Oxfordshire, UK). Fluo-4 AM was excited at 490 nm (bandwidth 15 nm) with Polychrome V at 30% light intensity (TILL Photonics, Pittsburgh, USA) with an exposure time of 20 ms; the emission was collected at 510–560 nm. The recording was done at a rate of 2 frames per second with the binning of 2 × 2 with Live Acquisition software v. 2.4.0.13 (FEI Munich GmbH), at least for 5 min (600 frames) time. From each individual fluorescence time-series a corresponding brightfield images were taken to assess cell morphology.

The mechanical stimulation was induced by touching single hESC-RPE cell with a glass micropipette during the recording of Ca^2+^ activity, similarly as described above. The micropipette was made from a glass capillary (Biomedical Instruments, Zollnitz, Germany; 0.86 9 1.50 9 80 mm) with a Narishige pipette puller. The micropipette was mounted on a Luigs & Neumann SM 5–9 vertical microinjection system (Luigs & Neumann, Ratingen, Germany) and intermittently lowered to touch a target cell membrane [25]. In samples with fresh wounds (9 days + 15 min and 28 days + 15 min), the site for mechanical stimulation was chosen to be in the vicinity to the wound edge. In samples with healed or partially healed wounds (9 days + 7 days and 28 days + 7 days), the stimulated cell belonged to the healed area.

All experiments were performed at RT at least 4 times. For each wounded area, the non-wounded area from the same coverslip was used as a control. The presented data are combined from Regea08/017 and Regea08/023 hESC-RPEs.

### Ca^2+^ imaging data analysis

The recorded time-series were analyzed with our in-house Matlab (R2013 A, Mathworks) algorithms and Fiji [32].

The cells on fluorescence time-series images were identified as described in [22]. At first cell centers were detected from averaged fluorescence images in Matlab with in-house algorithm developed for this purpose. Thereafter, cells were approximated as fixed-sized circles in Fiji [32]. The cells with immature, fusiform morphology, this fitting was done manually in Fiji. Next, average fluorescence kinetics from each detected cell at every recorded time point was extracted in Fiji and exported to Matlab for further analysis.

In Matlab, the fluorescence kinetics from single cells was corrected for background fluorescence and smoothed with a moving average algorithm with the 5 points span. Then, baselines in the beginning and in the end of a recording were automatically detected for each cell, and the fluorescence curves were corrected for bleaching and normalized to initial baseline levels. Finally, the peaks in the fluorescence curves were automatically detected. The amplitude of peak threshold was set to 1.1 (10% higher than the baseline). The cell identification and fluorescence curves analysis algorithms together with Matlab codes were similar to the ones used in Abu Khamidakh et al. [22]. The fluorescence kinetics from single cells was corrected for background fluorescence and smoothed with a moving average algorithm with the 5 points span. The fluorescence values were corrected for bleaching with the normalizing value achieved by comparing baselines in the beginning and in the end of a recording from each cell. Finally, the peaks in the fluorescence curves were automatically detected. The amplitude of peak threshold was set to 1.1 (10% higher than the baseline).

The spontaneous activity of hESC-RPEs was assessed as the percentage of cells with spontaneous [Ca^2+^]_i_ increases (%RC—percentage of responsive cells). In wounded samples, the cellular monolayer in the field of view was divided into several areas: 1—“Wound” area included the cells in the healed part of a monolayer (analyzed from samples that were fully or partially healed during the 7-day healing time), 2—“Area 1” included cells directly adjacent to the wound (within a distance of 40–60 μm), 3—“Area 2” consisted of cells directly adjacent to “Area 1” (40–60 μm distance), 4—“Area 3” included cells that immediately followed “Area 2” (40–60 μm distance). The cells belonging to each area were automatically detected with an in-house algorithm developed for this purpose.

The spreading of mechanically induced Ca^2+^ waves was estimated by identifying cells that were participated in the wave. Any two cells were considered to propagate a wave, if they had fluorescence peaks with a peak time difference of less than 4 s, and the distance between these cells was less than 2–3 characteristic cell sizes [22]. Only the cells that had a fluorescence peak and which were connected to the cell/site of mechanical stimulation via other cells having fluorescence peak were considered to be involved in a wave. Cell clusters with small Ca^2+^ waves that were disconnected from the site of mechanical stimulation were considered as spontaneous Ca^2+^ waves. The extent of Ca^2+^ wave spreading was assessed by calculating a total number of cells propagating a Ca^2+^ wave and by estimating the area in the field of view that was covered by the wave. The presented data are combined from Regea08/017 and Regea08/023 hESC-RPEs.

### Ethical issues

University of Tampere has the approval of the National Authority for Medicolegal Affairs Finland (TEO) to study human embryos (Dnro 1426/32/300/05) and a supportive statement of the Ethical Committee of the Pirkanmaa Hospital District to derive, culture, and differentiate hESC lines from surplus human embryos (R05116). No new lines were derived for this study.

### Statistics

The statistical significance between healed cell areas, and differences in wound healing time and speed was done with two-sample unpaired Student’s *t* test. The number of cells involved in a mechanically induced Ca^2+^ wave, or area of Ca^2+^ wave spreading in different groups was done with Mann–Whitney test. The p < 0.05 was considered statistically significant. The results were expressed as mean ± standard error of mean (SEM). Number of samples is referred to as n_s_ in the figure legends.

## Results

### Morphology and differentiation status of wounded and intact hESC-RPE cells

**Fig. 1:**
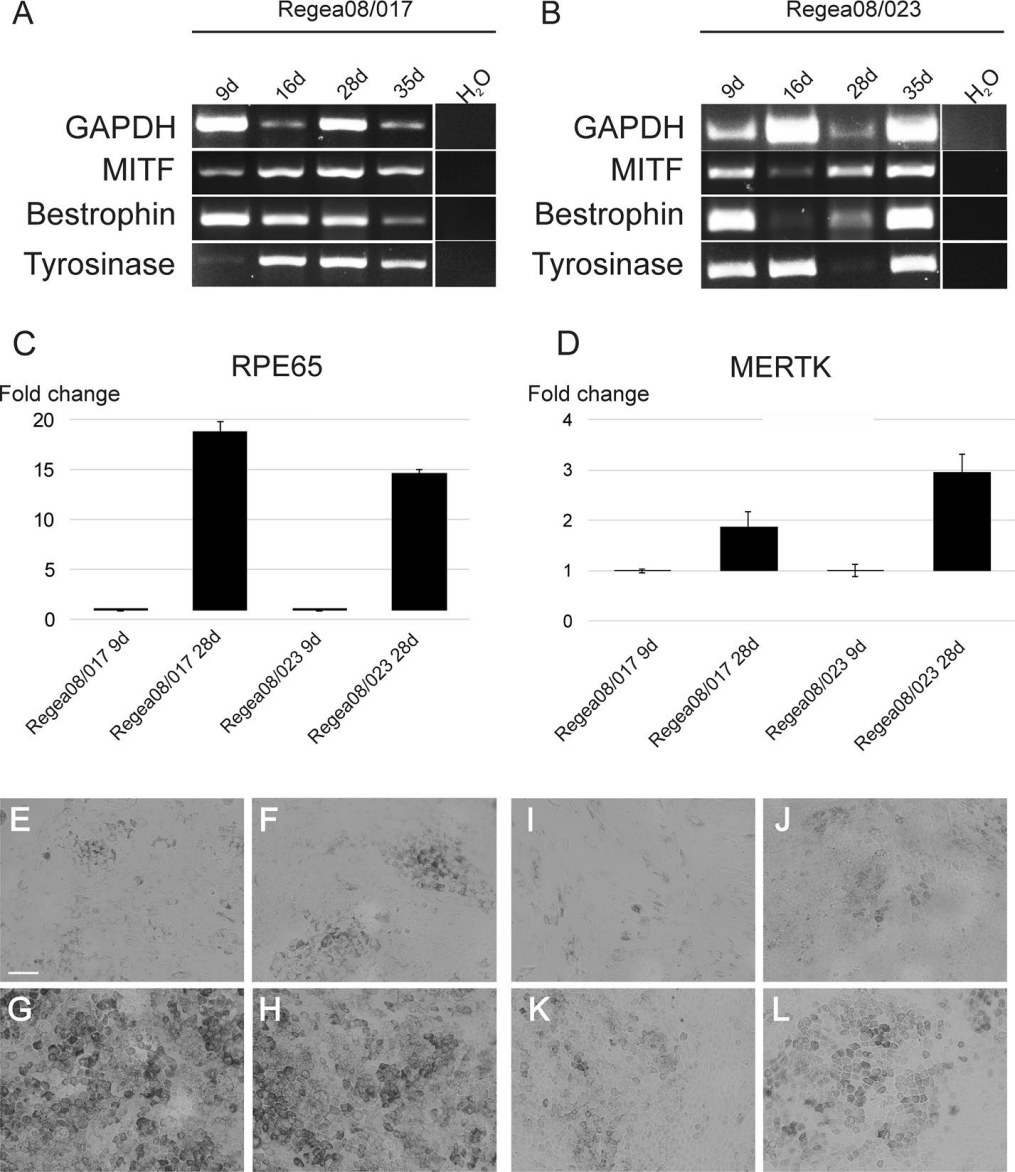
Gene expression analysis and morphology of hESC-RPE cells. Gene expression of a house-keeping gene GAPDH, and RPE-specific marker genes MITF, bestrophin and tyrosinase were assessed with reverse transcription–polymerase chain reaction (RT-PCR) from Regea08/017 (**A**) and Regea08/023 (**B**) hESC-RPE cells cultured for 9, 16, 28, and 35 days. Water was used as negative control. Relative gene expression of mature RPE markers, the retinal pigment epithelium specific protein 65 kDa (RPE65) (**C**) and receptor tyrosine kinase MerTK (MERTK) (**D**) of 9-day and 28-day cultured non-wounded (i.e. control) samples. The Ct values of target genes were approximately 30. A bright filed image of Regea08/017 hESC-RPEs cultured for 9 days (**E**), 16 days (**F**), 28 days (**G**), and 35 days (**H**). Bright filed image of Regea08/023 hESC-RPEs cultured for 9 days (**I**), 16 days (**J**), 28 days (**K**), and 35 days (**L**). Scale bar 50 μm

Differentiation status of 9-, 16-, 28-, and 35-day-cultured hESC-RPE cells was evaluated with RT-PCR. The samples from both Regea08/017 and Regea08/023 cell lines expressed RPE-specific markers *MITF*, *bestrophin* and *Tyrosinase* (Fig. 1A, B). The maturation status was further assessed with quantitative RT-PCR with two RPE specific genes. The expression of both *RPE65* and *MERTK* was very low (i.e. the target gene Ct:s were approximately 30), still there was a detectable increase in *RPE65* and *MERTK* mRNA expression when the 9 day and 28 day cultured samples were compared together. The morphology and pigmentation of representative 9-, 16-, 28-, and 35-day-cultured cells from both cell lines is represented on bright field images in Fig. 1E–H (Regea08/017) and Fig. 1I–L (Regea08/023).

**Fig. 2:**
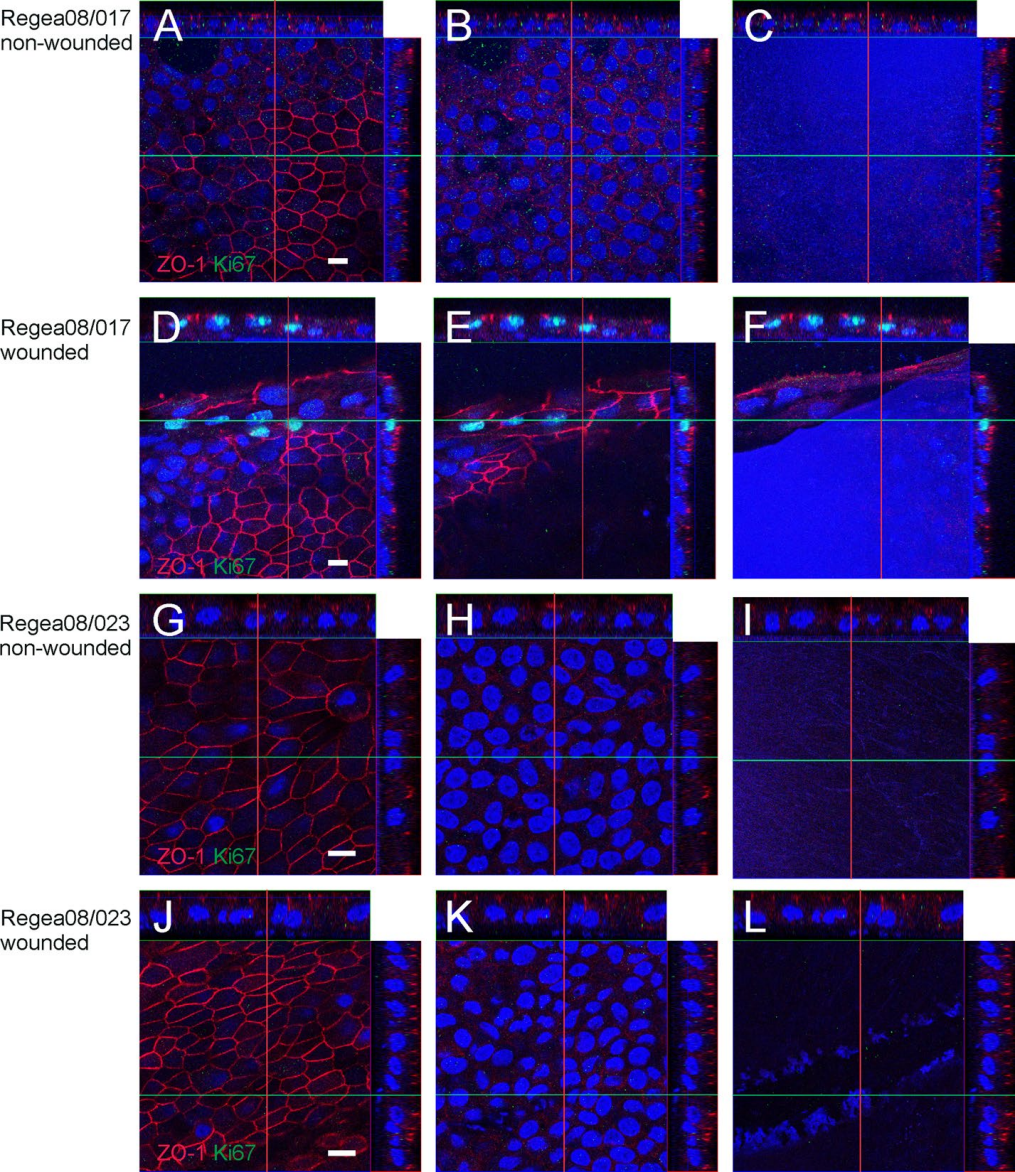
Morphology and polarity of hESC-RPE cultures. Localization of tight junction protein ZO-1 (red) and Ki67-positive proliferative cells (green) in control and wounded Regea08/017 and Regea08/023 hESC-RPEs. Nuclei were counterstained with DAPI (blue). Non-wounded area of Regea08/017 hESC-RPEs cultured for 35 days (**A**–**C**). Regea08/017 hESC-RPEs wounded on day 28 of culture and allowed to heal for 7 days, 35 days of culture in total (**D**–**F**). Non-wounded area of Regea08/023 hESC-RPEs cultured for 35 days (**G**–**I**). Regea08/023 hESC-RPEs wounded on day 28 of culture and allowed to heal for 7 days, 35 days of culture in total (**J**–**L**). On the left row are the Z-stack from the apical side, in the middle from the nuclear level, and on the right row from the basal side of the culture. The cross-hairs of Z-plane indicators are intensified for clarity. Scale bar 10 μm

The cultures were labelled with the tight junction protein ZO-1 antibody to visualize the cell confluence and polarization (Fig. 2). Indirect immunofluorescence staining showed that Regea08/017 hESC-RPE cells expressed ZO-1 on cell junctions in control non-wounded samples (Fig. 2A–C), as well as in wounded and healed samples (Fig. 2D–F), although the cells surrounding the wound still had elongated i.e. fusiform shaped morphology. The Regea08/023 hESC-RPE samples (Fig. 2G–L) were similar to the Regea08/023 hESC-RPEs.

The presence of proliferating cells was evaluated by labeling cultures with Ki67 antibody. There were no Ki67 positive cells in Regea08/017 hESC-RPE the control cultures (Fig. 2A–C). In wounded Regea08/017 cultures that were partially healed on day 7 after wounding, there were only few Ki67 positive cells (Fig. 2D–F) which located close to the edge, but not on the 1^st^ rim of cells lining the wound. In the Regea08/023 monolayer, there were no dividing, i.e. Ki67 positive cells either in control or wounded and 7 days healed samples were found (Fig. 2J–l).

**Fig. 3:**
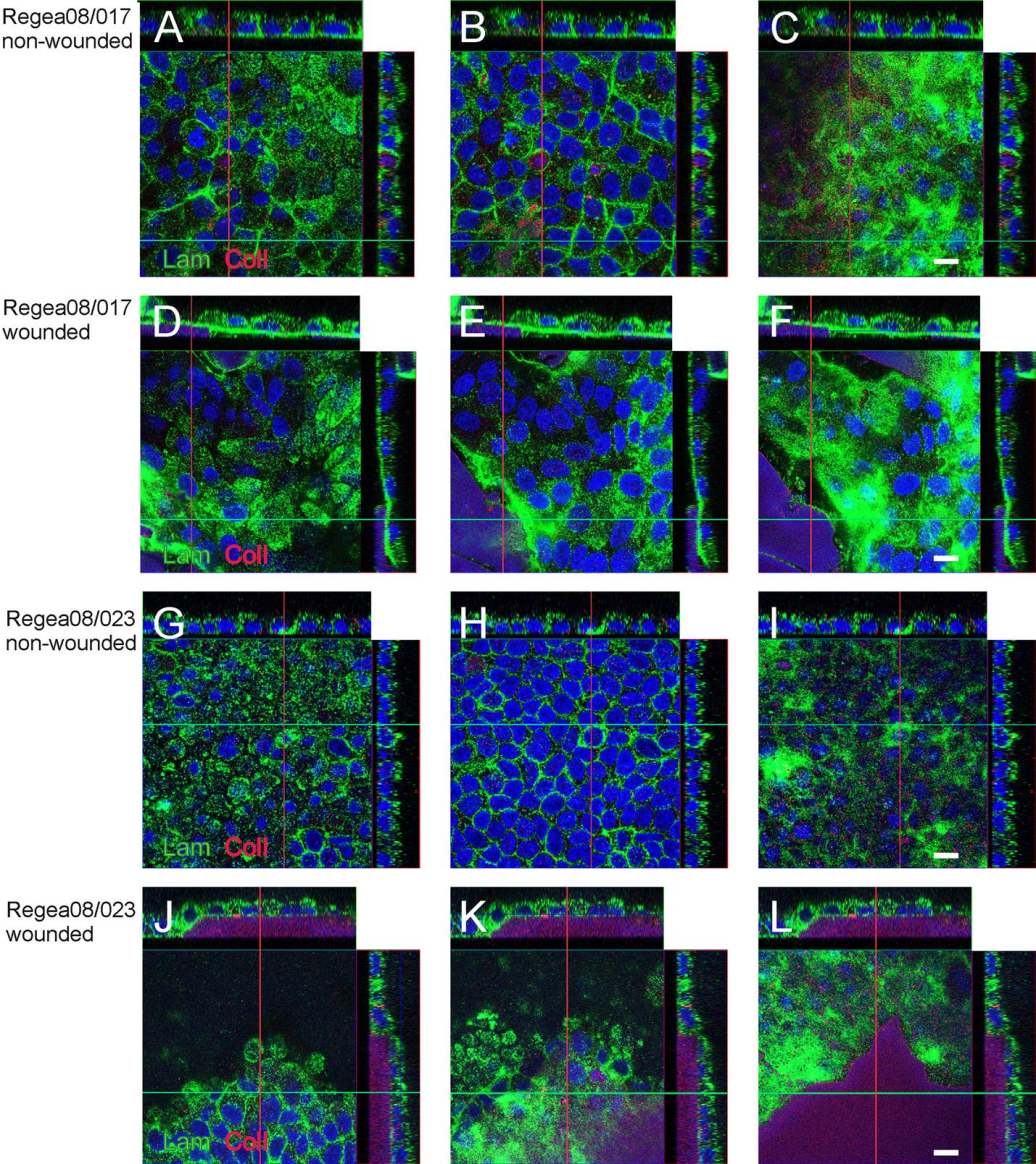
Extracellular protein localization in hESC-RPE cultures. Localization of extracellular matrix protein collagen I (red) and laminin (green) in control and wounded Regea08/017 and Regea08/023 hESC-RPEs. Nuclei were counterstained with DAPI (blue). Non-wounded area of Regea08/017 hESC-RPEs cultured for 16 days (**A**–**C**). Regea08/017 hESC-RPEs cultured for 9 days, wounded and imaged 7 days after wounding, cultured for 16 days in total (**D**–**F**). Non-wounded area of Regea08/023 hESC-RPEs cultured for 16 days (**G**–**I**). Regea08/023 hESC-RPEs wounded on day 9 of culture and allowed to heal for 7 days, cultured for 16 days in total (**J**–**L**). On the left row are the Z-stack from the apical side, in the middle from the nuclear level, and on the right row from the basal side of the culture. The cross-hairs of Z-plane indicators are intencified for clarity. Scale bar: 10 μm

Because migration and wound healing depends on the cell substrata, [33] we next assessed the localization of laminin and ColI. We saw that Regea08/017 hESC-RPEs produced laminin in control cultures (Fig. 3A–C). After wounding cells produced a thin layer of ColI (Fig. 3D–F). In the wounded and healed culture of Regea08/023 hESC-RPE cells produced a clearly marked layer of ColI (Fig. 3J–L). The deposition of COLI labelling close to the substrata (arrows on the orthopanels), and low background labelling in other areas of the cells are presented in Additional file 1: Fig S1.

**Fig. 4:**
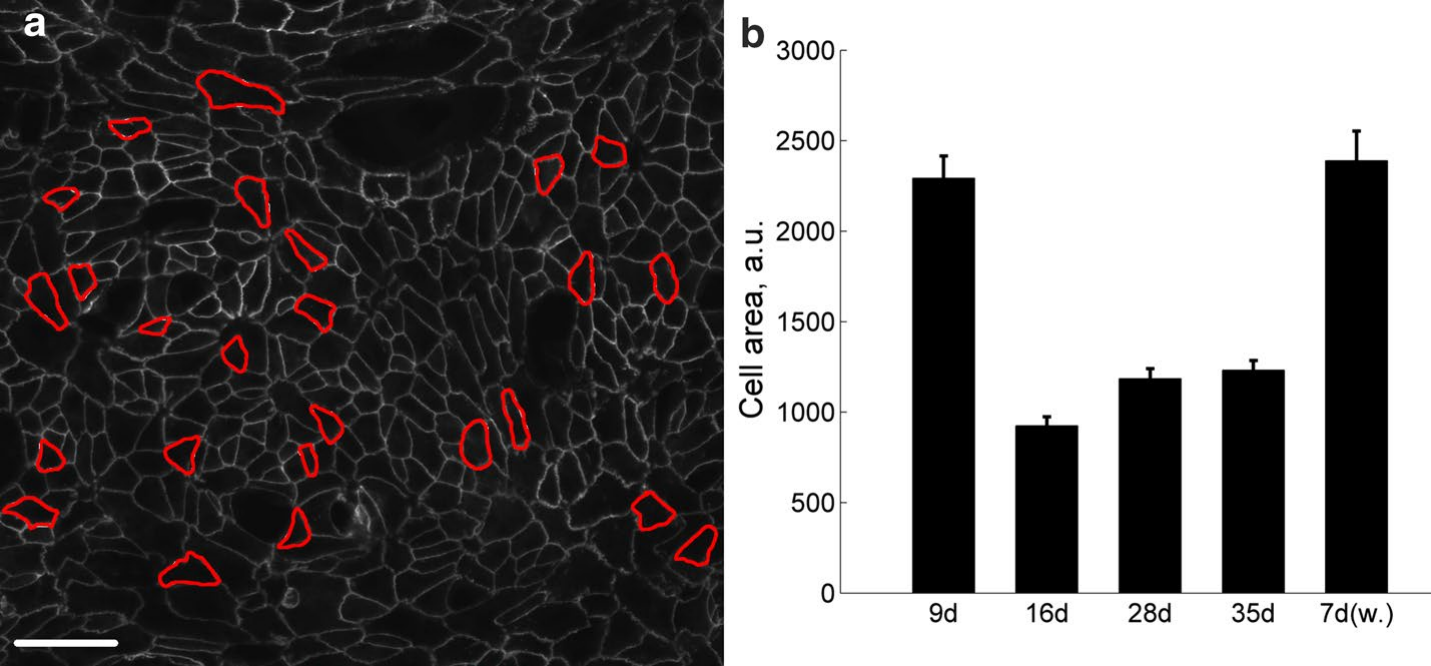
Cell size differences in 9- to 35-day-cultured hESC-RPE. Cell area was analyzed by manual cell boarder segmentation indicated by red lines (**a**). Scale bar: 50 μm. Analysis of cell areas (**b**) of hESC-RPEs cultured for 9 days (n_s_ = 4), 16 days (n_s_ = 6), 28 days (n_s_ = 6) or 35 days (n_s_ = 5), and 28-day-cultured hESC-RPEs, wounded and visualized 7 days after wounding (7 days) (n_s_ = 2). The presented data are combined from Regea08/017 and Regea08/023 hESC-RPEs

Because the culture time influences the morphology of the cells, we analyzed cell sizes by measuring the cell areas in 9-, 16-, 28-, and 35-day-cultured hESC-RPEs and in cells inside healed wounds in 28 days + 7 days samples. An example of manual cell border segmentation is presented in Fig. 4a. Figure 4b indicates that cells wounded on the day 28 of culture have the same size inside the healed wounds after 7 days as 9-day-cultured cells. In the Table 3 are all *p*-values of presented datasets.

**Table 3:** *p*-values calculated with the unpaired Student’s two-sample t-test for datasets presented in Fig. 4

p	9 days	16 days	28 days	35 days	7 days w.
9 days	–				
16 days	2.33 × 10^−20^	–			
28 days	2.08 × 10^−14^	4.94 × 10^−04^	–		
35 days	1.44 × 10^−13^	3.48 × 10^−05^	0.541	–	
7 days w.	0.636	3.30 × 10^−15^	4.67 × 10^−11^	1.94 × 10^−10^	–

Samples are abbreviated as follows: “9 days”—9-day-cultured; “16 days”—16-day-cultured; “28 days”—28-day-cultured; “35 days”—35-day-cultured; “7 days w.”—7-day-cultured cells within wounds made on day 28

### Wound healing speed of hESC-RPEs

**Fig. 5:**
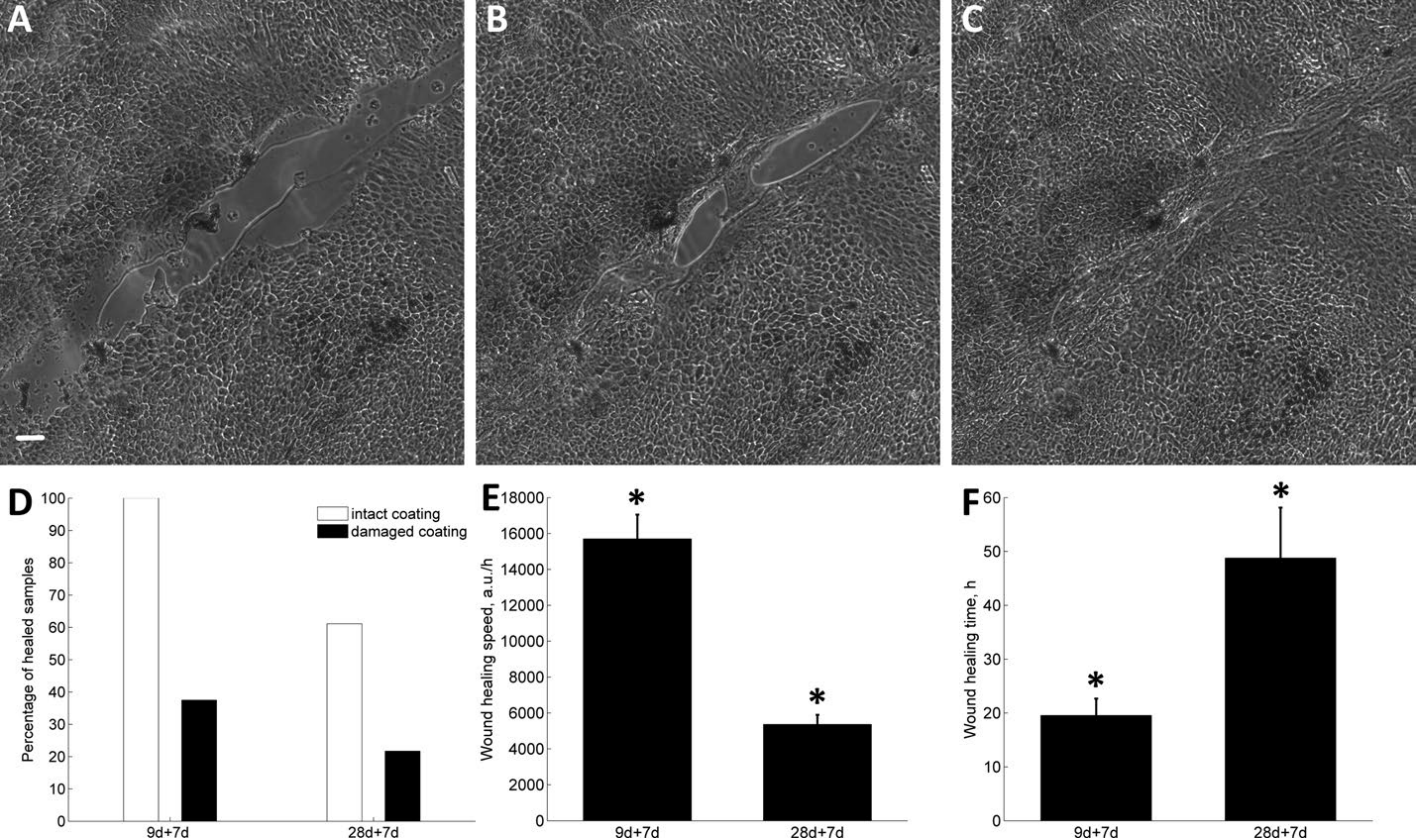
Wound healing speed and time. Bright field image of Regea08/023 hESC-RPEs wounded on day 28 of culture, then healed for 7 days, visualized immediately after wounding (**A**), 58 h after wounding (**B**), and 118 h after wounding (**C**). Scale bar: 50 μm. Percentage of completely healed samples (white bar—intact coating; black bar—damaged coating) wounded on day 9 and healed for 7 days (coating intact, n_s_ = 16; coating damaged, n_s_ = 8) or wounded on day 28 and healed for 7 days (coating intact, n_s_ = 18; coating damaged, n_s_ = 23) (**D**). Wound healing speed during a 7-day follow-up of 9-(n_s_ = 16) or 28-day-cultured (n_s_ = 11) hESC-RPEs (**E**). Wound healing time during a 7-day follow-up of 9-(n_s_ = 16) or 28-day-cultured (n_s_ = 11) hESC-RPE cells (**F**). The presented data are combined from Regea08/017 and Regea08/023 hESC-RPEs

To assess wound healing capabilities of hESC-RPE cells, the 9- and 28-day-cultured cells were followed for 7–8 days post-wounding. Both wounded 9- and 28-day-cultured hESC-RPEs were able to heal by filling denuded areas with cells (Fig. 5A–C). Samples, where ColIV coating was damaged during wounding, had significantly poorer ability to heal (Fig. 5D). When no visible ColIV damage occurred during the wounding process, 100% of samples wounded on day 9 of culture and 61% of samples wounded on day 28 were able to heal. The monolayers wounded on day 9 healed almost 3 times faster compared to those wounded on day 28 (*p *= 2 × 10^−6^) (Fig. 5E). The full healing time of wounded 9-day-cultured hESC-RPEs was 20 ± 3 h, while 28-day-cultured cells healed 2.5-fold slower with a healing time of 49 ± 9 h (*p *= 0.002). (Fig. 5F).

### Spontaneous [Ca^2+^]_i_ increases in wounded hESC-RPEs

**Fig. 6:**
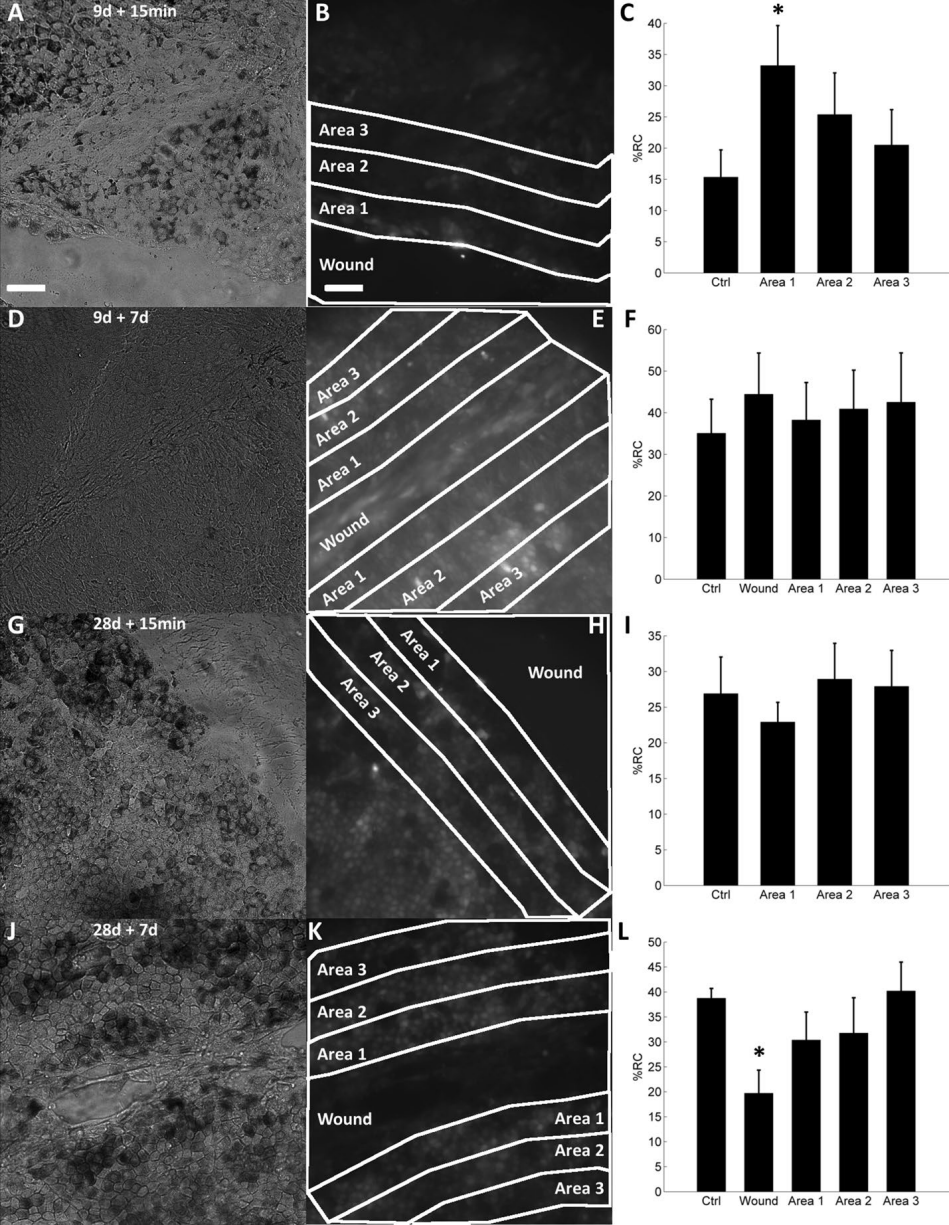
Percentage of cells with spontaneous [Ca^2+^]_i_ increases (%RC) in wounded hESC-RPEs at different areas related to the wound location. A bright field image of 9-day-cultured, wounded hESC-RPEs, visualized immediately after wounding (**A**). A fluorescence image of the same sample as in **A** loaded with Ca^2+^-sensitive indicator Fluo-4 AM (**B**). The wound area (“wound”) and adjacent areas (“Area 1”, “Area 2”, “Area 3”) are marked with white lines. Analyzed percentage of responsive cells (%RC) in non-wounded control (n_s_ = 8), and area 1 (n_s_ = 8), area 2 (n_s_ = 8) and area 3 (n_s_ = 8) (**C**). Statistical significance: *p* < 0.05 is marked with Asterisk. A bright field image of 9-day-cultured then wounded hESC-RPEs, visualized 7 days after wounding (**D**). A fluorescence image of the same sample as in D loaded with Ca^2+^-sensitive indicator fluo-4 AM (**E**). The wound area and adjacent areas are marked with white lines. The %RC in non-wounded control (n_s_ = 8), wounded area (n_s_ = 8) and area 1 (n_s_ = 8), area 2 (n_s_ = 8), and area 3 (n_s_ = 6) (**F**). A bright field image of 28-day-cultured and wounded hESC-RPEs, visualized immediately after wounding (**G**). A fluorescence image of the same sample as in G loaded with Ca^2+^-sensitive indicator Fluo-4 AM (**H**). The wound area and adjacent areas are marked with white lines. The %RC in non-wounded control (n_s_ = 8) and area 1 (n_s_ = 8), area 2 (n_s_ = 8), and area 3 (n_s_ = 8) (**I**). A bright field image of 28-day-cultured then wounded hESC-RPEs, visualized 7 days after wounding (**J**). A fluorescence image of the same sample as in J loaded with Ca^2+^-sensitive indicator Fluo-4 AM (**K**). The wound area and adjacent areas are marked with white lines. The %RC in non-wounded control (n_s_ = 4), wounded area (n_s_ = 4) and area 1 (n_s_ = 4), area 2 (n_s_ = 4), and area 3 (n_s_ = 4) (**L**). The presented data are combined from Regea08/017 and Regea08/023 hESC-RPEs

Then, the effect of wounding on spontaneous transient [Ca^2+^]_i_ increases was evaluated. The samples of 9 d + 15 min (Fig. 6A–C), 9 days + 7 days (Fig. 6D–F), 28 days + 15 min (Fig. 6G–I), and 28 days + 7 days (Fig. 6J–L) samples are in Fig. 6. On the left are the representative bright field images, in the middle are the corresponding fluorescence images of the cells in the same field of view loaded with Fluo-4 AM are shown, and on the right are the analysed areas, where the percentage of the cells with spontaneous [Ca^2+^]_i_ increases (%RC) were compared. In 9-day-cultured hESC-RPE cells with fresh wounds (9 days + 15 min samples), %RC in the area closest to the wound edge (“area 1”) was more than twice higher compared to control (%RC in area 1 = 33.2 ± 6.4%;  %RC in control = 15.3 ± 4.4%; *p *= 0.028) (Fig. 6C). The 9 days + 7 days (Fig. 6F) and 28 days + 15 min samples (Fig. 6I) did not have statistically significant changes in %RC in any of the investigated areas. Healed or partially healed hESC-RPEs that were wounded on day 28 of culture and then allowed to heal for 7 days (28 days + 7 days samples) had almost twofold decreased  %RC inside the healed wound compared to the control (%RC in the healed wound area = 19.7 ± 4.6; %RC in control = 38.7 ± 2.0%; *p *= 0.029) (Fig. 6L).

### Mechanically induced Ca^2+^ waves in wounded hESC-RPEs

Next, the effect of wounding on the propagation of intercellular Ca^2+^ waves induced by mechanical stimulation was assessed. Mechanical stimulation of a single cell in a hESC-RPE monolayer resulted in a [Ca^2+^]_i_ increase in the stimulated cell that propagated in a wave-like manner to neighboring cells in 9 days + 15 min, 9 days + 7 days, 28 days + 15 min, and 28 days + 7 days samples.

**Fig. 7:**
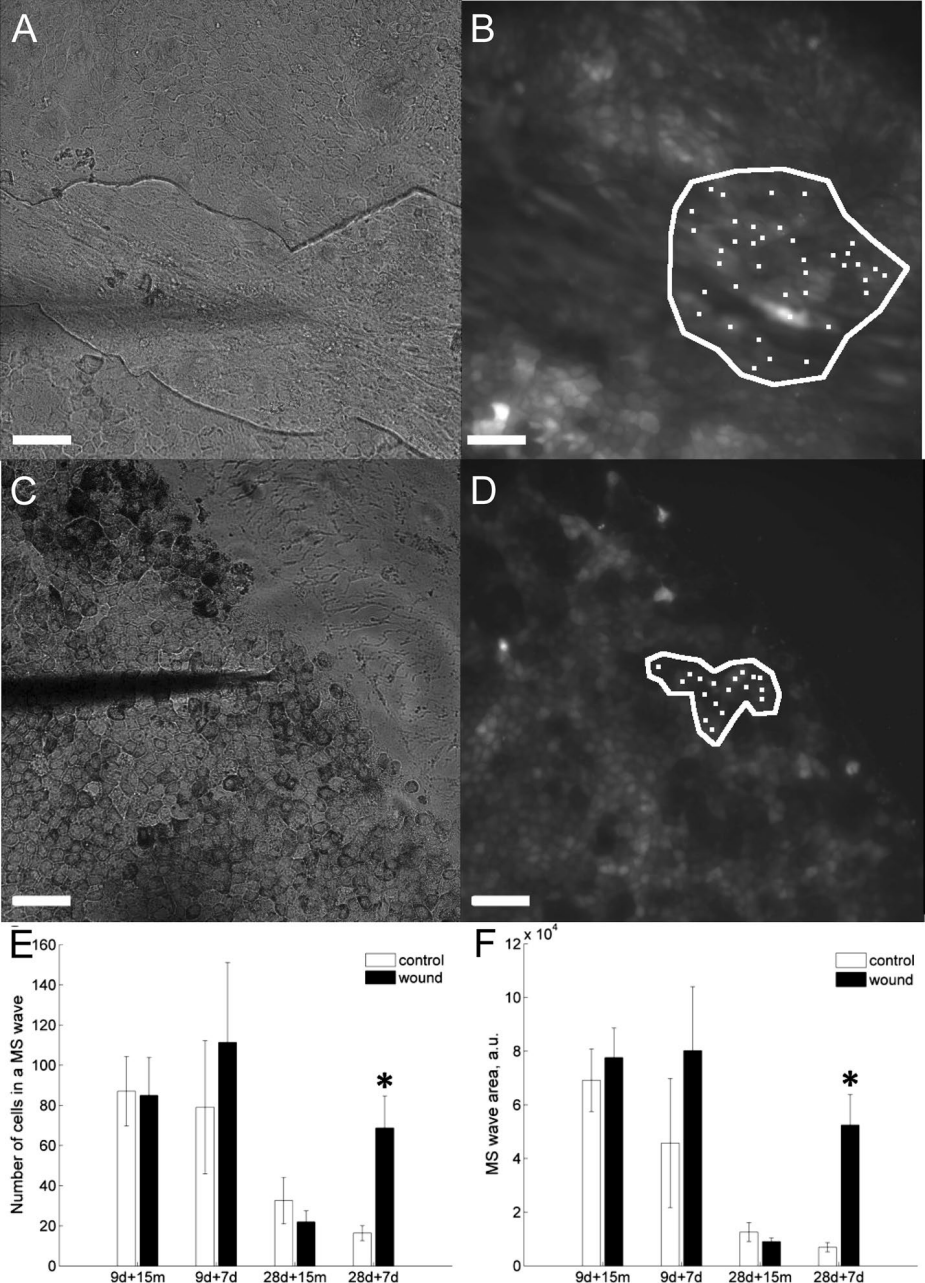
Ca^2+^ wave propagation in wounded hESC-RPEs after mechanical stimulation. A bright field image of a hESC-RPE monolayer (9d + 7d) (**A**). The dark shadow of micropipette lowered towards the cell to perform mechanical stimulation can be seen. Fluorescence image of the same culture as in A loaded with fluorescent Ca^2+^ sensitive dye Fluo-4 AM that reflects [Ca^2+^]_i_ concentration in the cytoplasm (**B**). A white line indicates the area of the monolayer that responded to single-cell mechanical stimulation. White dots indicate the cells that participate in a mechanically induced Ca^2+^ wave. Similarly a bright field image of a hESC-RPE monolayer (28 days + 7 days) (**C**). The dark shadow of micropipette lowered towards the cell to perform mechanical stimulation can be seen. Fluorescence image of the same culture as in A loaded with fluorescent Ca^2+^ sensitive dye Fluo-4 AM that reflects [Ca^2+^]_i_ concentration in the cytoplasm (**D**). A white line indicates the area of the monolayer that responded to single-cell mechanical stimulation. White dots indicate the cells that participate in a mechanically induced Ca^2+^ wave. Number of hESC-RPE cells participating in a mechanically induced intercellular Ca^2+^ wave in wounded 9-day-cultured cells followed for 15 min (ctrl, n_s_ = 7; wounded, n_s_ = 7), or for 7 days (ctrl, n_s_ = 7; wounded, n_s_ = 7). The mechanically induced intercellular Ca^2+^ wave in wounded 28-day-cultured cells followed for 15 min (ctrl, n_s_ = 8; wounded, n_s_ = 8), or for 7 days (ctrl, n_s_ = 6; wounded, n_s_ = 7). **E** White bar—control; black bar—wound edge or healed wound. Statistical significance: *p *< 0.05 is indicated with Asterisk. Area of a mechanically induced intercellular Ca^2+^ wave spreading in wounded 9-day-cultured cells followed for 15 min (ctrl n_s_ = 7; wounded, n_s_ = 7), or for 7 days (ctrl, n_s_ = 7; wounded, n_s_ = 7). The mechanically induced intercellular Ca^2+^ wave spreading in wounded 28-day-cultured cells followed for 15 min (ctrl, n_s_ = 8; wounded n_s_ = 8), or for 7 days (ctrl, n_s_ = 6; wounded, n_s_ = 7). **F** White bar—control; black bar—wound edge or healed wound. Statistical significance: *p *< 0.05 is indicated with Asterisk. The presented data are combined from Regea08/017 and Regea08/023 hESC-RPEs

The wounded hESC-RPE monolayer (9 days + 7 days) and a shadow of a micropipette used for mechanical stimulation is represented in Fig. 7A and the fluorescence image of the same culture loaded with Ca^2+^-sensitive dye in the same field of view is shown in Fig. 7B, see also corresponding Additional file 2: Video S1. The white polygon indicates the area of wave spreading, and cells indicated with white dots are the cells participating in the wave propagation. A representative brightfield images with a wounded hESC-RPE monolayer and a shadow of a micropipette used for mechanical stimulation is represented in Fig. 7C (the 28 days + 7 days) and the fluorescence image of the same culture loaded with Ca^2+^-sensitive dye in the same field of view is shown in Fig. 7D, see also corresponding Additional file 3: Video S2. The latter figure also indicates the area of Ca^2+^ wave spreading (white polygon) and the cells participating in the wave propagation (white dots).

Samples that were cultured for 28 days prior to wounding and later allowed to heal for 7 days (28 days + 7 days samples) showed a 4.6-fold higher number of cells involved in Ca^2+^ wave spreading within a healed area compared to control (79 ± 19 cells in wounded area and 17 ± 6 cells in control area; *p *= 0.0047) (Fig. 7E). Furthermore, in these samples Ca^2+^ waves covered 8.9-fold larger areas compared to control (*p *= 0.0023) (Fig. 7F).

In 9 d + 15 min, 9 days + 7 days, and 28 days + 15 min samples Ca^2+^ waves inside or close to the wounded areas propagated in the same manner as in non-wounded controls (Fig. 7E, F.

## Discussion

The complex RPE-derived wound healing process in AMD is weakly understood. In a recent study the healing of scratch wounded H9 hESC-RPE cells has been shown to have partial wound closure within 30 days [12]. Ca^2+^ has an important role in the wound healing of epithelial cells but less is known about cellular Ca^2+^ dynamics implicated in the response to fresh wounds and wound healing of RPE. Here, we analyzed spontaneous and mechanically induced [Ca^2+^]_i_ increases of cells in freshly wounded and healed hESC-RPE monolayers at two different maturation stages. There are great expectations of hESC-RPE as becoming cell source for cell replacement therapy [34–36]. To understand the integration of surgical transplants, it is essential to study the wound healing mechanisms of the hESC-RPEs.

For RPE cells, the ability to heal scratch wounds has been demonstrated in different in vitro and in vivo models, e.g. in [5, 7–9, 12]. For hESC-RPE cells, Schwartz et al. showed that, in vitro, their attachment to substrata strongly depends on cell differentiation stage: the lighter pigmented cells attach and proliferate better than heavily pigmented cells. Thus, it was proposed that less mature cells may provide better results for transplantation studies [37]. In line with the latter, we found that wound healing capacity of our hESC-RPEs depended on cell culture time: cells wounded after 9-days of culture showed a higher percent of healed samples and faster healing speed compared to those wounded on 28-day of culture. Substratum is crucial for wound healing: the percentage of healed samples with damaged collagen IV coating was much lower compared to that with intact coating in hESC-RPE cells wounded on both day 9 and day 28 after plating. This is in line with the knowledge that cells in general prefer the extracellular matrix substrata and that the plain Ormocomp coating does not support hESC-RPE cell spreading [29]. In addition, with confocal orthoimages, we showed that within 7 days the cells had produced new extracellular matrix layer with laminin and collagen I to the site of the wound and had migrated on top of it. In confocal analyses this layer appeared to be thicker than the extracellular matrix prior to the wounding.

In epithelial monolayers, several rows of cells behind the wound edge participate in wound healing, pushing the monolayer towards the denuded area [38–40]. In chick model of small wounds, the RPE cells stretch from wound edges towards each other and then fill the wound without proliferation [7]. But, if the wound is wide, RPE cells start proliferating to seal it [7]. In accordance with these data, we saw hESC-RPE cells which were proliferating during wound healing, but there were no proliferating cells after the wound closure. In cultures with proliferating cells, the Ki-67 positive cells located few cell layers away from the wound edge.

The scratch-wounding of a cellular monolayer is known to trigger an intercellular Ca^2+^ wave that spreads several rows away from the leading wound edge [18, 41–43]. For example in human corneal epithelial cells, it has been shown that ATP released at the time of injury serves as an early signal that enables activation of wound healing processes [44]. In our 9-day cultured hESC-RPEs, after wounding we detected the elevated percentage of cells with spontaneous [Ca^2+^]_i_ increases close to the wound edge. Thus, in this study, we could also observe distinct behavior of the cells near the denuded area in terms of Ca^2+^ dynamics compared to the rest of the monolayer. We can hypothesize that the initial Ca^2+^ wave which occurs at the time of wounding affects the Ca^2+^ dynamics of the cells close to the wound edge to promote the healing.

In freshly wounded 28-day-cultured cells, the percentage of cells with spontaneous [Ca^2+^]_i_ increases was the same as in control, regardless of the distance to the wound edge. We hypothesize that 28-day-cultured hESC-RPEs are not able to make their spontaneous activity any higher, as previously we have shown that the percentage of cells with spontaneous [Ca^2+^]_i_ increases does not elevate further after day 28 of culture [22].

Here, the hESC-RPEs wounded on day 28 of culture and then allowed to heal for 7 days, had lower percentage of cells with spontaneous [Ca^2+^]_i_ increases inside the healed area. This finding is in accordance with our previously published observation that the percentage of cells with spontaneous [Ca^2+^]_i_ increases raises during maturation in hESC-RPE cells [22]. Thus, cells with shorter culture time within the healed wounds have lower spontaneous Ca^2+^ activity compared to surrounding cells, which have been cultured for a longer period of time.

Mechanical stimulation has been shown to induce intercellular Ca^2+^ waves in RPE [22–25]. In rat RPE, the mechanically induced intercellular Ca^2+^ waves spread to up to 3 cell tiers away from the site of mechanical stimulation [23, 24], whereas in human ARPE-19 cell line, such waves are more intense, covering up to 14 cell layers [25]. Our previous studies show that in hESC-RPE cells, the spreading of mechanically induced Ca^2+^ waves strongly depends on cell culture time. In cells cultured for a longer period of time (28 days), the Ca^2+^ waves propagate to only few cells away from the stimulation site, similarly to the waves in rat RPE. On the other hand, the cells cultured for a shorter period of time (9 days) respond with wide-spreading Ca^2+^ waves, similarly to ARPE-19 [22].

In the current study, we demonstrated that in hESC-RPEs wounded on day 28 of culture and then allowed to heal for 7 days, mechanical stimulation resulted in wide-spreading intercellular Ca^2+^ waves, when a cell inside the healed wound had been stimulated. Inside the healed wounds, the cells have less mature morphology compared to the non-wounded surroundings, and indeed, such a strong response to mechanical stimulation is typical for immature hESC-RPEs.

On the other hand, control non-wounded areas exhibited more mature morphology, and the wave spreading was restricted to only few cells that is typical for more mature hESC-RPE cells, as we have shown in our previous study [22]. In contrast, the cells wounded on day 9 of culture and then allowed to heal for 7 days, did not show significant differences in the mechanically stimulated Ca^2+^ wave spreading in the healed areas and in control. The cells within the healed areas had similar morphology as the surrounding cells, and the Ca^2+^ wave spreading pattern corresponded to immature hESC-RPE cells. In addition, when cells were wounded on day 9 of culture and then healed for 7 days, the cells inside the healed area did not show significant difference in the percentage of cells with spontaneous [Ca^2+^]_i_ activity compared to the surroundings. Thus, we can speculate that if there is no big difference in maturation status of the cells within the wound and in the surroundings, the cells can adapt to share similar Ca^2+^ dynamics behavior.

## Conclusions

We have shown here that the effect of wounding on Ca^2+^ dynamics of hESC-RPE monolayers depends on cell culture time: 9-day-cultured freshly wounded cells have elevated amount of cells with spontaneous [Ca^2+^]_i_ increases in vicinity to the wound compared to control, whereas 28-day-cultured freshly wounded cells had similar Ca^2+^ dynamics around the wound edge as in other areas. Most importantly, we have shown that 28-day-cultured, wounded and thereafter 7-day-healed areas resemble the behavior of 9-day-cultured cells both in Ca^2+^ dynamics and cell morphology. In addition, we have demonstrated that cells cultured for a shorter period of time heal faster than cells cultured for a longer period of time. This acquired knowledge about Ca^2+^ dynamics in hESC-RPE cells is important for understanding the fundamental mechanisms of RPE wound healing that can lead to new insights in AMD pathological process and therapy.

## Additional files

**Additional file 1: Fig S1.** Confocal images of non-wounded (A) and wounded (B) Regea08/017 hESC-RPE cultures in which COLI is shown in red and nuclei in blue. As seen from the ortho-sections on the right and above, the COLI is concentrated close to the substrata. This is indicated also with white arrows. The background labeling, which would be visible is other areas of the cell, is very low. Scale bars are 10 µm.

**Additional file 2: Video S1.** Calcium wave in hESC-RPE monolayer (9d + 7d) followed for 300 s after mechanical stimulation. The video corresponds Fig. 7A, B. Prior the stimulation hESC-RPEs (9d + 7d) were loaded with fluorescent Ca^2+^ sensitive dye Fluo-4 AM that reflects [Ca^2+^]_i_ concentration in the cytoplasm. The site of mechanical stimulation is marked with white an arrow. Mechanical stimulation of a single cell in a hESC-RPE monolayer resulted in a [Ca^2+^]_i_ increase, seen as an increase in fluorescent signal, in the stimulated cell that propagates in a wave-like manner to neighbouring cells.

**Additional file 3: Video S2.** The video corresponds Fig. 7C, D. Calcium wave in hESC-RPE monolayer (28d + 7d) followed for 300 s after mechanical stimulation. Prior the stimulation hESC-RPEs were loaded with fluorescent Ca^2+^ sensitive dye Fluo-4 AM that reflects [Ca^2+^]_i_ concentration in the cytoplasm. The site of mechanical stimulation is marked with white an arrow. Mechanical stimulation of a single cell in a hESC-RPE monolayer resulted in a [Ca^2+^]_i_ increase, seen as an increase in fluorescent signal, in the stimulated cell that propagates in a wave-like manner to neighbouring cells.

## Abbreviations

RPE: retinal pigment epithelium
hESCs: human embryonic stem cells
