# Supplementary figures and images for: Wound healing of human embryonic stem cell-derived retinal pigment epithelial cells is affected by maturation stage
Source: Biomed Eng Online. 2018 Jul 31;17:102. doi: 10.1186/s12938-018-0535-z (PMC6069779; doi:10.1186/s12938-018-0535-z)

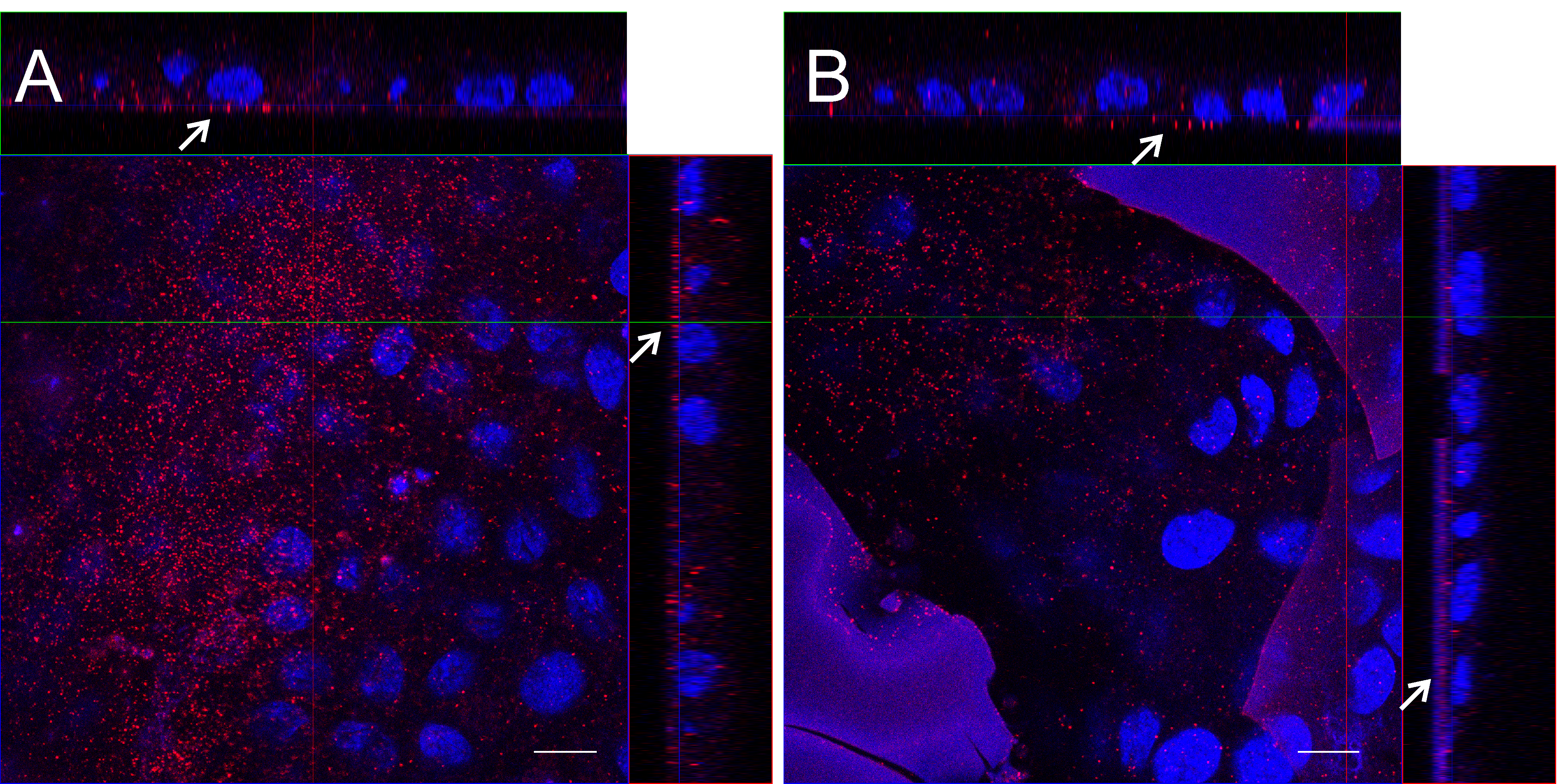

Supplement: Supplementary file 1 — Additional file 1: Fig S1. Confocal images of non-wounded (A) and wounded (B) Regea08/017 hESC-RPE cultures in which COLI is shown in red and nuclei in blue. As seen from the ortho-sections on the right and above, the COLI is concentrated close to the substrata. This is indicated also with white arrows. The background labeling, which would be visible is other areas of the cell, is very low. Scale bars are 10 µm. [file 12938_2018_535_MOESM1_ESM.tif]
